# Tris[2-(2-pyridylimino­meth­yl)phenol­ato(0.67−)]europium(III) nitrate

**DOI:** 10.1107/S1600536809017206

**Published:** 2009-05-29

**Authors:** Qi-Hua Zhao, Hong-Yan Chen, Li-Nan Li, Ming-Jin Xie

**Affiliations:** a School of Chemical Science and Technology, Key Laboratory of Medicinal Chemistry for Natural Resources, Ministry of Education, Yunnan University, Kunming 650091, People’s Republic of China

## Abstract

The title compound, [Eu(C_12_H_9.33_N_2_O)_3_]NO_3_, was obtained by the reaction of Eu(NO_3_)·3H_2_O and the Schiff base ligand 2-(2-pyridylimino­meth­yl)phenol. The Eu atom is located on a threefold rotation axis and is nine-coordinated by three tridentate Schiff base ligands in a distorted tricapped trigonal-prismatic geometry. The O atom at the phenol hydr­oxy group is partially deprotonated and the H atoms are modelled with one-third occupancy according to the space group *R* 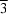. Offset face-to-face π–π [centroid–centroid distance = 3.886 (3) Å] and edge-to-face C—H⋯π inter­actions are found between adjacent mol­ecules. An intra­molecular O—H⋯N hydrogen bond is also present.

## Related literature

For the synthesis, see: Sreenivasulu *et al.* (2005 ▶); Henry *et al.* (2008 ▶). For related structures, see: Li & Zhang (2004 ▶); You *et al.* (2004 ▶).
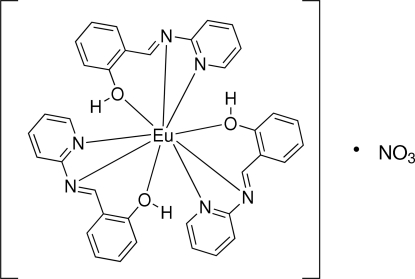


## Experimental

### 

#### Crystal data

[Eu(C_12_H_9.33_N_2_O)_3_]NO_3_
*M* *_r_* = 806.61
Hexagonal, 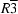

*a* = 14.0398 (12) Å
*c* = 28.509 (5) Å
*V* = 4866.7 (11) Å^3^
*Z* = 6
Mo *K*α radiation
μ = 1.99 mm^−1^
*T* = 293 K
0.21 × 0.15 × 0.10 mm

#### Data collection

Bruker APEXII CCD area-detector diffractometer
Absorption correction: multi-scan (*SADABS*; Sheldrick, 1996 ▶) *T*_min_ = 0.706, *T*_max_ = 0.819
10540 measured reflections
2599 independent reflections
1711 reflections with *I* > 2σ(*I*)
*R*_int_ = 0.092

#### Refinement

*R*[*F* ^2^ > 2σ(*F* ^2^)] = 0.054
*wR*(*F* ^2^) = 0.128
*S* = 1.00
2599 reflections
155 parameters
H atoms treated by a mixture of independent and constrained refinement
Δρ_max_ = 1.11 e Å^−3^
Δρ_min_ = −0.90 e Å^−3^

### 

Data collection: *APEX2* (Bruker, 2004 ▶); cell refinement: *SAINT* (Bruker, 2004 ▶); data reduction: *SAINT*; program(s) used to solve structure: *SHELXS97* (Sheldrick, 2008 ▶); program(s) used to refine structure: *SHELXL97* (Sheldrick, 2008 ▶); molecular graphics: *SHELXTL* (Sheldrick, 2008 ▶); software used to prepare material for publication: *SHELXTL*.

## Supplementary Material

Crystal structure: contains datablocks I, global. DOI: 10.1107/S1600536809017206/bq2130sup1.cif

Structure factors: contains datablocks I. DOI: 10.1107/S1600536809017206/bq2130Isup2.hkl

Additional supplementary materials:  crystallographic information; 3D view; checkCIF report

## Figures and Tables

**Table 1:** Selected geometric parameters (Å, °)

Eu1—O1	2.334 (4)
Eu1—N2	2.539 (5)
Eu1—N1	2.680 (5)

**Table 2:** Hydrogen-bond geometry (Å, °)

*D*—H⋯*A*	*D*—H	H⋯*A*	*D*⋯*A*	*D*—H⋯*A*
O1—H1*B*⋯N2	0.89 (14)	2.09 (14)	2.783 (6)	134 (11)
C12—H12*A*⋯*Cg*1^iii^	0.93	2.88	3.788 (9)	167

Symmetry code: (iii) 
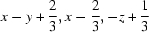
. *Cg*1 is the centroid of the C7–C12 benzene ring.

**Fig. 1.**
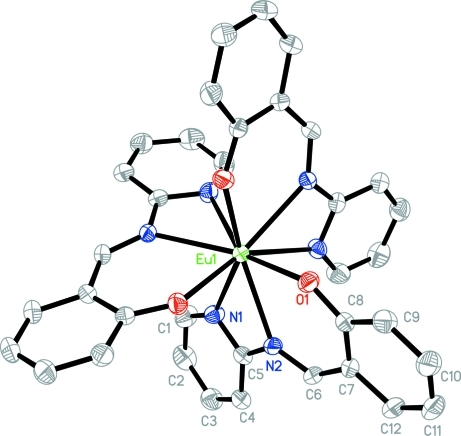
The molecular structure of the title compound with the atom-numbering scheme. Displacement ellipsoids are drawn at the 30% probability level.

## supplementary crystallographic information

### Comment

During the last decades, considerable amount of work was devoted to the synthesis, structure and properties of transition metal complexes derived from Schiff bases because of their potential applications in catalysis and enzymatic reactions, magnetism and molecular architecture (Henry *et al.* 2008; Li & Zhang, 2004). Herein, we report the Schiff base complex mentioned in the title by solvent evaporation method (Sreenivasulu *et al.*, 2005).

As shown in Fig. 1, the central Eu of the title compound is nine-coordinated. The coordination environment is defined by six N atoms and three O atoms from the three different *N*-Salicylidene-2-aminopyride ligands. (You *et al.*, 2004). The bond length of Eu(1)—N(1) (2.681 (5) Å) and Eu(1)—N(2) (2.540 (5) Å) are longer than Eu(1)—O(1) (2.332 (4) Å). The bond angle of O(1)#1-Eu(1)—N(2)#2 (69.37 (14)°) is larger than N(2)#2-Eu(1)—N(1) (51.11 (15)°).

In one schiff base ligand, all of the atoms are almost in one plane. The most evident distortion is associated with the C12 atom, which is 0.2017 (3)Å away from the mean plane. Meanwhile, the two aromatic rings of the same ligand form a dihedral angle of 14.072 (4)°, and between every two neighbour ligands coordinated to the Eu, the schiff bases appear an angle of 80.768 (3), 80.292 (4), 80.933 (3)°, respectively. The phenyl ring and pyridine ring of adjacent molecules exist the offset face-to-face pi-pi stacking interactions, with a distance of 3.886 (3)Å (13.245 (4)°) and the edge-to-face C—H-pi interactions were founded between the two phenyl rings of adjacent molecules with the distance of 3.785 (5) Å. The intramolecular hydrogen-bonding was also found between O1 and N2 atoms with the N···O separation of 2.783 (6) Å.

### Experimental

All chemicals used (reagent grade) were commercially available. Salicylaldehyde (0.122 g, 1 mmol) and 2-aminomethylpyridine (0.108 g, 1 mmol) were dissolved in ethanol (5 ml) respectively at room temperature. Then the two solutions were mixed and stirred slowly for about 30 min. Finally, the yellow ligand was synthesized. Then Eu(NO_3_)_2_.3H_2_O (0.400 g, 1 mmol) in ethanol (5 ml) was added to it with stirring homogeneously. Yellow crystals suitable for X-ray ananlysis were obtained by slow evaporation at room temperature over several days.

### Refinement

H atoms bonded to C atoms were calculated geometrically and allowed to ride on the C atoms with distance restraints of C—H = 0.93Å and *U*_iso_(H) = 1.2*U*_eq_(C). The H atom bonded to atom O1 was located in a difference map and refined with the distance restraints O—H = 0.89 (14)Å and the H atoms was modelled with one-third occupancy.

### Crystal data

**Table:**

[Eu(C_12_H_9.33_N_2_O)_3_]NO_3_	*F*(000) = 2424
*M**_r_* = 806.61	*D*_x_ = 1.651 Mg m^−^^3^
Hexagonal, *R*3	Mo *K*α radiation, λ = 0.71073 Å
Hall symbol: -R 3	θ = 1.9–28.4°
*a* = 14.0398 (12) Å	µ = 1.99 mm^−^^1^
*c* = 28.509 (5) Å	*T* = 293 K
*V* = 4866.7 (11) Å^3^	Block, yellow
*Z* = 6	0.21 × 0.15 × 0.10 mm

### Data collection

**Table:**

Bruker APEXII 1K CCD area-detector diffractometer	2599 independent reflections
Radiation source: fine-focus sealed tube	1711 reflections with *I* > 2σ(*I*)
graphite	*R*_int_ = 0.092
φ and ω scans	θ_max_ = 28.4°, θ_min_ = 1.8°
Absorption correction: multi-scan (*SADABS*; Sheldrick, 1996)	*h* = −13→18
*T*_min_ = 0.706, *T*_max_ = 0.819	*k* = −18→18
10540 measured reflections	*l* = −37→35

### Refinement

**Table:**

Refinement on *F*^2^	Primary atom site location: structure-invariant direct methods
Least-squares matrix: full	Secondary atom site location: difference Fourier map
*R*[*F*^2^ > 2σ(*F*^2^)] = 0.054	Hydrogen site location: inferred from neighbouring sites
*wR*(*F*^2^) = 0.128	H atoms treated by a mixture of independent and constrained refinement
*S* = 1.00	*w* = 1/[σ^2^(*F*_o_^2^) + (0.059*P*)^2^] where *P* = (*F*_o_^2^ + 2*F*_c_^2^)/3
2599 reflections	(Δ/σ)_max_ < 0.001
155 parameters	Δρ_max_ = 1.11 e Å^−^^3^
0 restraints	Δρ_min_ = −0.90 e Å^−^^3^

### Special details

**Table:**

Geometry. All e.s.d.'s (except the e.s.d. in the dihedral angle between two l.s. planes) are estimated using the full covariance matrix. The cell e.s.d.'s are taken into account individually in the estimation of e.s.d.'s in distances, angles and torsion angles; correlations between e.s.d.'s in cell parameters are only used when they are defined by crystal symmetry. An approximate (isotropic) treatment of cell e.s.d.'s is used for estimating e.s.d.'s involving l.s. planes.
Refinement. Refinement of *F*^2^ against ALL reflections. The weighted *R*-factor *wR* and goodness of fit *S* are based on *F*^2^, conventional *R*-factors *R* are based on *F*, with *F* set to zero for negative *F*^2^. The threshold expression of *F*^2^ > σ(*F*^2^) is used only for calculating *R*-factors(gt) *etc*. and is not relevant to the choice of reflections for refinement. *R*-factors based on *F*^2^ are statistically about twice as large as those based on *F*, and *R*- factors based on ALL data will be even larger.

### Fractional atomic coordinates and isotropic or equivalent isotropic displacement parameters (Å^2^)

**Table:**

	*x*	*y*	*z*	*U*_iso_*/*U*_eq_	Occ. (<1)
Eu1	1.0000	0.0000	0.16281 (2)	0.0373 (2)	
O1	1.0522 (4)	0.1495 (3)	0.21296 (15)	0.0466 (11)	
H1B	1.108 (11)	0.139 (10)	0.206 (4)	0.05 (3)*	0.33
O2	0.977 (3)	0.068 (2)	0.3090 (13)	0.558 (19)	
N1	1.1365 (4)	0.0071 (4)	0.09543 (18)	0.0450 (12)	
N2	1.1941 (4)	0.1521 (4)	0.14418 (17)	0.0404 (12)	
N3	1.0000	0.0000	0.3159 (6)	0.142 (7)	
C1	1.1473 (6)	−0.0492 (6)	0.0603 (2)	0.062 (2)	
H1A	1.0877	−0.1170	0.0520	0.075*	
C2	1.2458 (7)	−0.0091 (7)	0.0357 (3)	0.068 (2)	
H2A	1.2515	−0.0495	0.0111	0.081*	
C3	1.3364 (6)	0.0926 (6)	0.0483 (2)	0.0623 (19)	
H3A	1.4029	0.1202	0.0324	0.075*	
C4	1.3251 (5)	0.1502 (5)	0.0842 (2)	0.0505 (16)	
H4A	1.3841	0.2173	0.0936	0.061*	
C5	1.2233 (5)	0.1064 (5)	0.1066 (2)	0.0453 (15)	
C6	1.2517 (5)	0.2543 (5)	0.1551 (2)	0.0460 (16)	
H6A	1.3151	0.2975	0.1377	0.055*	
C7	1.2259 (5)	0.3067 (5)	0.1920 (2)	0.0404 (14)	
C8	1.1301 (5)	0.2504 (5)	0.2202 (2)	0.0398 (14)	
C9	1.1213 (5)	0.3104 (6)	0.2584 (2)	0.0536 (17)	
H9A	1.0619	0.2753	0.2787	0.064*	
C10	1.1979 (6)	0.4187 (6)	0.2661 (3)	0.070 (2)	
H10A	1.1888	0.4552	0.2914	0.084*	
C11	1.2886 (6)	0.4749 (6)	0.2370 (3)	0.070 (2)	
H11A	1.3391	0.5488	0.2420	0.084*	
C12	1.3017 (5)	0.4186 (5)	0.2009 (3)	0.0581 (18)	
H12A	1.3626	0.4553	0.1814	0.070*	

### Atomic displacement parameters (Å^2^)

**Table:**

	*U*^11^	*U*^22^	*U*^33^	*U*^12^	*U*^13^	*U*^23^
Eu1	0.0340 (2)	0.0340 (2)	0.0438 (3)	0.01701 (11)	0.000	0.000
O1	0.041 (2)	0.040 (2)	0.055 (3)	0.018 (2)	0.008 (2)	−0.001 (2)
O2	0.46 (3)	0.34 (3)	1.02 (6)	0.30 (2)	−0.05 (5)	0.03 (4)
N1	0.040 (3)	0.047 (3)	0.045 (3)	0.019 (3)	0.005 (2)	−0.006 (2)
N2	0.033 (3)	0.036 (3)	0.050 (3)	0.016 (2)	0.002 (2)	−0.006 (2)
N3	0.180 (12)	0.180 (12)	0.065 (9)	0.090 (6)	0.000	0.000
C1	0.066 (5)	0.054 (4)	0.060 (5)	0.024 (4)	0.004 (4)	−0.018 (4)
C2	0.086 (6)	0.087 (6)	0.048 (4)	0.055 (5)	0.000 (4)	−0.014 (4)
C3	0.061 (5)	0.069 (5)	0.063 (5)	0.036 (4)	0.008 (4)	−0.001 (4)
C4	0.047 (4)	0.053 (4)	0.056 (4)	0.029 (3)	0.000 (3)	0.001 (3)
C5	0.041 (4)	0.049 (4)	0.052 (4)	0.027 (3)	0.001 (3)	0.000 (3)
C6	0.034 (3)	0.035 (3)	0.062 (4)	0.012 (3)	0.001 (3)	0.000 (3)
C7	0.040 (3)	0.033 (3)	0.047 (4)	0.018 (3)	−0.002 (3)	−0.003 (3)
C8	0.038 (3)	0.039 (3)	0.046 (4)	0.022 (3)	−0.003 (3)	0.000 (3)
C9	0.049 (4)	0.060 (4)	0.053 (4)	0.028 (4)	−0.001 (3)	−0.018 (3)
C10	0.060 (5)	0.061 (5)	0.086 (6)	0.029 (4)	−0.009 (4)	−0.035 (4)
C11	0.057 (5)	0.042 (4)	0.098 (6)	0.016 (4)	−0.004 (4)	−0.021 (4)
C12	0.043 (4)	0.042 (4)	0.078 (5)	0.013 (3)	−0.004 (4)	−0.011 (4)

### Geometric parameters (Å, °)

**Table:**

Eu1—O1	2.334 (4)	C1—C2	1.395 (10)
Eu1—O1^i^	2.334 (4)	C1—H1A	0.9300
Eu1—O1^ii^	2.334 (4)	C2—C3	1.403 (10)
Eu1—N2^i^	2.539 (5)	C2—H2A	0.9300
Eu1—N2^ii^	2.539 (5)	C3—C4	1.361 (9)
Eu1—N2	2.539 (5)	C3—H3A	0.9300
Eu1—N1^ii^	2.680 (5)	C4—C5	1.397 (9)
Eu1—N1^i^	2.680 (5)	C4—H4A	0.9300
Eu1—N1	2.680 (5)	C6—C7	1.430 (8)
Eu1—C5^ii^	3.154 (6)	C6—H6A	0.9300
Eu1—C5^i^	3.154 (6)	C7—C12	1.412 (8)
Eu1—C5	3.154 (6)	C7—C8	1.420 (8)
O1—C8	1.302 (7)	C8—C9	1.418 (8)
O1—H1B	0.89 (14)	C9—C10	1.372 (9)
O2—N3	1.167 (16)	C9—H9A	0.9300
N1—C1	1.330 (8)	C10—C11	1.389 (10)
N1—C5	1.353 (8)	C10—H10A	0.9300
N2—C6	1.285 (7)	C11—C12	1.365 (9)
N2—C5	1.411 (7)	C11—H11A	0.9300
N3—O2^i^	1.167 (16)	C12—H12A	0.9300
N3—O2^ii^	1.167 (16)		
			
O1—Eu1—O1^i^	86.41 (15)	O1^i^—Eu1—H1B	101 (4)
O1—Eu1—O1^ii^	86.41 (15)	O1^ii^—Eu1—H1B	71 (3)
O1^i^—Eu1—O1^ii^	86.41 (15)	N2^i^—Eu1—H1B	102 (4)
O1—Eu1—N2^i^	80.87 (15)	N2^ii^—Eu1—H1B	140 (3)
O1^i^—Eu1—N2^i^	69.51 (15)	N2—Eu1—H1B	52 (4)
O1^ii^—Eu1—N2^i^	153.28 (16)	N1^ii^—Eu1—H1B	167 (3)
O1—Eu1—N2^ii^	153.28 (16)	N1^i^—Eu1—H1B	92 (3)
O1^i^—Eu1—N2^ii^	80.87 (15)	N1—Eu1—H1B	102 (4)
O1^ii^—Eu1—N2^ii^	69.51 (15)	C5^ii^—Eu1—H1B	166 (4)
N2^i^—Eu1—N2^ii^	115.74 (7)	C5^i^—Eu1—H1B	97 (3)
O1—Eu1—N2	69.51 (15)	C5—Eu1—H1B	77 (4)
O1^i^—Eu1—N2	153.28 (16)	C8—O1—Eu1	142.2 (4)
O1^ii^—Eu1—N2	80.87 (15)	C8—O1—H1B	83 (8)
N2^i^—Eu1—N2	115.74 (8)	Eu1—O1—H1B	68 (8)
N2^ii^—Eu1—N2	115.74 (7)	C1—N1—C5	118.6 (6)
O1—Eu1—N1^ii^	151.37 (16)	C1—N1—Eu1	144.0 (4)
O1^i^—Eu1—N1^ii^	86.02 (16)	C5—N1—Eu1	97.4 (4)
O1^ii^—Eu1—N1^ii^	120.57 (15)	C6—N2—C5	121.9 (5)
N2^i^—Eu1—N1^ii^	70.61 (15)	C6—N2—Eu1	134.4 (4)
N2^ii^—Eu1—N1^ii^	51.09 (15)	C5—N2—Eu1	102.1 (3)
N2—Eu1—N1^ii^	120.68 (15)	O2^i^—N3—O2	117.2 (12)
O1—Eu1—N1^i^	86.02 (16)	O2^i^—N3—O2^ii^	117.2 (12)
O1^i^—Eu1—N1^i^	120.57 (15)	O2—N3—O2^ii^	117.2 (12)
O1^ii^—Eu1—N1^i^	151.37 (16)	N1—C1—C2	121.5 (7)
N2^i^—Eu1—N1^i^	51.09 (15)	N1—C1—H1A	119.2
N2^ii^—Eu1—N1^i^	120.68 (15)	C2—C1—H1A	119.2
N2—Eu1—N1^i^	70.61 (15)	C1—C2—C3	119.6 (6)
N1^ii^—Eu1—N1^i^	74.31 (17)	C1—C2—H2A	120.2
O1—Eu1—N1	120.57 (15)	C3—C2—H2A	120.2
O1^i^—Eu1—N1	151.37 (16)	C4—C3—C2	118.8 (7)
O1^ii^—Eu1—N1	86.02 (16)	C4—C3—H3A	120.6
N2^i^—Eu1—N1	120.68 (15)	C2—C3—H3A	120.6
N2^ii^—Eu1—N1	70.61 (15)	C3—C4—C5	118.7 (6)
N2—Eu1—N1	51.09 (15)	C3—C4—H4A	120.7
N1^ii^—Eu1—N1	74.31 (17)	C5—C4—H4A	120.7
N1^i^—Eu1—N1	74.31 (17)	N1—C5—C4	122.8 (6)
O1—Eu1—C5^ii^	168.10 (15)	N1—C5—N2	109.3 (5)
O1^i^—Eu1—C5^ii^	81.98 (16)	C4—C5—N2	127.9 (6)
O1^ii^—Eu1—C5^ii^	95.44 (15)	N1—C5—Eu1	57.4 (3)
N2^i^—Eu1—C5^ii^	92.55 (15)	C4—C5—Eu1	175.7 (5)
N2^ii^—Eu1—C5^ii^	25.94 (15)	N2—C5—Eu1	51.9 (3)
N2—Eu1—C5^ii^	122.40 (15)	N2—C6—C7	125.0 (6)
N1^ii^—Eu1—C5^ii^	25.18 (15)	N2—C6—H6A	117.5
N1^i^—Eu1—C5^ii^	97.61 (16)	C7—C6—H6A	117.5
N1—Eu1—C5^ii^	71.32 (15)	C12—C7—C8	119.7 (6)
O1—Eu1—C5^i^	81.98 (16)	C12—C7—C6	117.4 (6)
O1^i^—Eu1—C5^i^	95.44 (16)	C8—C7—C6	122.9 (5)
O1^ii^—Eu1—C5^i^	168.10 (15)	O1—C8—C9	119.5 (6)
N2^i^—Eu1—C5^i^	25.94 (15)	O1—C8—C7	124.2 (5)
N2^ii^—Eu1—C5^i^	122.40 (15)	C9—C8—C7	116.3 (5)
N2—Eu1—C5^i^	92.55 (15)	O1—C8—H1B	36 (5)
N1^ii^—Eu1—C5^i^	71.32 (15)	C9—C8—H1B	143 (5)
N1^i^—Eu1—C5^i^	25.18 (15)	C7—C8—H1B	94 (5)
N1—Eu1—C5^i^	97.61 (16)	C10—C9—C8	121.9 (7)
C5^ii^—Eu1—C5^i^	96.46 (15)	C10—C9—H9A	119.0
O1—Eu1—C5	95.44 (16)	C8—C9—H9A	119.0
O1^i^—Eu1—C5	168.10 (15)	C9—C10—C11	121.4 (7)
O1^ii^—Eu1—C5	81.98 (16)	C9—C10—H10A	119.3
N2^i^—Eu1—C5	122.40 (15)	C11—C10—H10A	119.3
N2^ii^—Eu1—C5	92.55 (15)	C12—C11—C10	118.2 (6)
N2—Eu1—C5	25.94 (15)	C12—C11—H11A	120.9
N1^ii^—Eu1—C5	97.61 (16)	C10—C11—H11A	120.9
N1^i^—Eu1—C5	71.32 (15)	C11—C12—C7	122.3 (7)
N1—Eu1—C5	25.18 (15)	C11—C12—H12A	118.8
C5^ii^—Eu1—C5	96.46 (15)	C7—C12—H12A	118.8
C5^i^—Eu1—C5	96.46 (15)		

Symmetry codes: (i) −*y*+1, *x*−*y*−1, *z*; (ii) −*x*+*y*+2, −*x*+1, *z*.

### Hydrogen-bond geometry (Å, °)

**Table:**

*D*—H···*A*	*D*—H	H···*A*	*D*···*A*	*D*—H···*A*
O1—H1B···N2	0.89 (14)	2.09 (14)	2.783 (6)	134 (11)
C12—H12A···Cg1^iii^	0.93	2.88	3.788 (9)	167

Symmetry codes: (iii) *x*−*y*+2/3, *x*−2/3, −*z*+1/3.
